# A System of Making Jacket Porcelain Crowns without Fusing

**Published:** 1916-01

**Authors:** L. E. Custer

**Affiliations:** Dayton, Ohio


### A SYSTEM OF MAKING JACKET PORCELAIN CROWNS WITHOUT FUSING.

BY L. E. CUSTER, A. M., D. D. S., DAYTON, OHIO.

Owing to the high degree of skill and patience required the jacket crown is only employed by a select few. This crown has certain advantages possessed by no other form of crown, which should bring it into more general use, and the message I bring is a system whereby the country dentist who has no electric current at his disposal, or a dentist who has not taken up dental ceramics, can, with the ordinary dental outfit, make a jacket crown which is practically as good, and indeed better in some respects than one baked by the dentist himself.

### ADVANTAGES OF THE PORCELAIN JACKET CROWN.

The jacket crown possesses certain features which easily place it at the head of all other forms of porcelain crowns. First, the strength and durability of this crown is testified to by every dentist who has made one. Dr. W. A. Capon of Philadelphia says, "After many years of experience with different kinds of porcelain jacket crowns, I am glad that I was fortunate enough to recognize their efficiency early in my practice. When a root has been crowned to death and considered only fit for extraction, a jacket crown will give it renewed life and vigor in the majority of cases, if it is decently firm in its socket."

Dr. Edward B. Spalding of Detroit says, "The all-porcelain jacket crown and its modifications have displaced all other forms of porcelain crowns in my practice. The gum tissue is always more healthy about a carefully fitted and flush joint than where a band is used."

Dr. George Schneider of Chicago says, "There are two vital points in favor of the jacket crown, namely, first it is not necessary to remove the natural crown in whole; second, you do not endanger the root by enlarging the canal for the retention of a post."

Dr. H. E. Jenkins of Ironton, Ohio, whom I have seen repeatedly drive a canine jacket crown of his own make through an inch pine board without damage to the crown, maintains and proves that it possesses strength above any other form of porcelain crown.

The strength of the jacket crown is due largely to the natural post of dentin within it, which is a part of the tooth itself. Where caries has left but little dentin, this is reinforced by a platinoiridium post occupying approximately the pulpal space of the tooth. We have never seen or heard of a root split under a jacket crown. This can not be said of any other form of porcelain crown.

A second advantage of the packet crown, as pointed out by Dr. Spalding, is that it makes a flush joint with the root at the cervix. A metal band with its uncertain fit is thus done away with.

The third advantage is the esthetic appearance of the completed crown. The entire crown itself performs the functions of a band, thus eliminating the unsightly metal band at the gum line.

OBJECTIONS.

The disadvantages of the jacket crown lie entirely in the technique of its construction. The operator must be skilled in the working of porcelain, and I know of no procedure in dentistry that requires so high a degree of skill and patience as the making of a porcelain jacket crown. The platinum coping requires skill and time in its formation, the selection and fusing of the proper shade of porcelain requires years of experience, and then often at the last minute the esthetic appearance of the whole appliance may be spoiled by overfusing. Another objection is the amount of time consumed in the baking method.

It is a system or procedure in which the objections just enumerated are overcome that I herewith present, and since I am the inventor of the first electric oven, it may seem strange that I advocate a method which does not require an oven, nor does it require any special instruments. It will also be noticed that many steps of the technique are old and more or less familiar to everyone.

### PREPARATION OF THE TOOTH.

The first step is the preparation of the tooth, the technique of which is practically the same as for a baked crown and quite simple, seldom requiring more than fifteen minutes. With a carborundum disk the sides of the tooth are first cut off. Then, with a wheel, the incisal end of enamel is removed approximately to the dentin.

The end should not be removed first, for it requires more grinding. Then, with the same or a smaller wheel the enamel is removed both labially and lingually and the corners rounded. The fourth step consists in squaring the shoulder just under the gum line, which is done with a small bell-shaped carborundum point. The final dressing is done with a flexible sandpaper disk and point.

### SELECTION AND PREPARATION OF CROWN.

A pinless crown of any make and of the size and color suitable for the case is now selected. The inside is hollowed out with small carborundum or diamond points, which operation requires about fifteen minutes. This, however, can be done by the assistant. I have myself bought a selection of biscuited crowns, hollowed them out with a large bur, and returned them to the manufacturer for final baking. I am told that if this procedure is taken up to any extent, ready-made jacket crowns will be put upon the market, thus saving the grinding-out of the crown.

The hollowed-out crown is now filled with soft, black wax, such as is used for carding teeth, warmed, and placed upon the stump of dentin. If the crown is dry and the stump wet, the wax will remain in the crown, and upon removal will show just where grinding will be necessary for a closer fit. This manipulation is repeated until the labial surface of the cervix of the crown passes under the gum margin.

SEALING OF THE JOINT BETWEEN CROWN AND ROOT.

We now come to the important step in the entire procedure, that is, the perfect sealing of the joint at the cervix between the crown and the root. Ney & Co. furnish a platinum and gold foil, equal parts, beaten down to a No. 10 foil. If the joint is fairly well fitted, without any space wider than thin pasteboard, a piece of this foil about one inch square for a lateral incisor, and larger for a central or cuspid, is crumpled into a rope. The ends are cut on the bias which, when brought together around the stump of the tooth, will overlap, and if annealed will cohere and facilitate manipulation. The crown is then put in place over the rope with firm pressure and light malleting. While the crown is held in place, a thin burnisher is passed entirely around the bulging washer, and the gold is condensed against and into the joint. The gold-and-platinum rope now forms a washer which perfectly fits the root on one side and the crown on the other. It is then removed, and placed in an investment of carborundum and alcohol in a smal fire-clay cup.

CARBORUNDUM AS AN INVESTMENT MATERIAL.

I here call attention, perhaps for the first time, to the use of carborundum such as is used in grinding automobile valves as an investment material in dentistry, because of its infusibility and high specific gravity. The carborundum packs closely around the washer, forming a firm support, and to some extent prevents warping of the metal. Although infusible, it appears to cohere when at a high heat, for a little difficulty is experienced in getting the washer out and breaking up the carborundum. It is far superior to silex when used for similar purposes, and may be used over and over again.

The washer is now placed in the investment at a slight angle, and by gentle tapping on the tray is allowed to settle until entirely embedded, excepting the lap joint. A small piece of coin gold, well boraxed with a saturated solution of borax and boracic acid, is placed on the exposed lap. The alcohol is burned off and a flame is directed at the cup from *below,* the object of this procedure being that, when the solder melts, the washer being hotter than the solder, the latter will be immediately drawn into and fill the washer. There will be no danger of melting the washer even when coin gold is used to solidify the same. This is because of the high percentage of platinum in the foil. In my first experiments I tried pure gold foil with a low-karat solder, but found the foil to be always warped and melted in the thin places even when as low as 10-karat solder was used.

### CEMENTING JACKET CROWN TO PLACE.

We now have a washer of high karat which fits the root on one side and the crown on the other. It is dressed on its periphery so as to be flush with the root and crown. The crown and washer are now to be set, and, for this purpose I wish to recommend the use of Tenacit, a translucent cement made by DeTrey Bros. for crown and bridge work. We are all aware that the silicious cements resist chemical action in gingival positions better than the oxyphosphate cements. This material, moreover, produces no shadow effects as does cement under thin porcelain. Tenacit adds to the lifelike appearance of the crown.

In order to prevent weeping of the gum tissue and secure perfect dryness, the gum is touched with a 20 per cent. solution of trichloracetic acid. This is a powerful astringent, and its effect will last amply long enough to remove the surplus Tenacit and cover with varnish.

### SECURING JOINT IN BADLY CARIOUS ROOTS.

The procedure just recited applies to those cases where the carious process has not extended beyond the gum margin, but in those cases where it has gone beyond the gum line to any extent at any one point, the metal washer must be thickened at that place. If the caries is not too deep, the ends of the rope need not be cut on the bias, but may simply overlap, which will give a double thickness at this point. In order to insure the coin-gold solder permeating between the different folds of the foil, three or four punctures at the lap are made with an ordinary pin or sharp-pointed instrument. When the decay is quite deep, extra pieces of foil are built up at such points.

### ADVANTAGES OF WRITER'S METHOD.

A crown made in the foregoing manner is, to my mind, in regard to security of attachment and seal against future decay, not only equal to a crown fused by the operator, but has four distinct advantages: (1) the selection of color is absolutely assured at the very outset. No vagaries of the fusing process enter into the final shade. The operator knows at once what the final appearance will be. Moreover, there will be no change of color in the future, as appears to take place in office-baked porcelain. While this is more frequently seen with inlays, largely owing to the fact that difference of color is more noticeable in contiguous surfaces, still it takes place to some extent in office-baked jacket crowns. There appears to be a bleaching effect in the course of time. Factory-baked procelain does not apparently change color. (2) The porcelain of a factory-baked crown is stronger than that made by the dentist, because the porcelain is condensed under considerable pressure, and is more evenly tempered when cooling. (3) While the crown may not fit as perfectly on the inside to the stump of dentin, depending upon how much time the operator devotes to this step, a closer sealing at the gum line, the *essential* step, is achieved by the metal washer than by a porcelain crown after the platinum coping has been stripped from within. The fit on the inside depends upon the amount of time the operator gives to this step, but in practice, the inside fit, when the crown is set with Tenacit, is quite immaterial. (4) A great saving in time is effected, for the entire operation seldom requires more than an hour for its completion.

### FURTHER APPLICATION OF THE GASKET METHOD.

Any thoughtful operator can easily see the application of the gasket method of cervical sealing to Logan and other fixed pin crowns, and he can also, by this method, as I have often done, make the washer perform the function of an intradental ring by cutting a groove in the end of the root half-way between the root-canal and the periphery. Moreover, by using a thicker rope of gold and one which is somewhat larger in diameter so as to bulge more at the joint, the bulging portion can be burnished so as to form a perfectly fitting band around the end of the root or crown, or both at the same time. Such a washer, when it is removed and each side is filled with plumbago paste, can be thickened to any extent with coin gold.

While it might seem to some that, if the thought of this paper were carried out, the highest grade of dental ceramic art would deteriorate, I have no such fears, for those who have become skilled in the making of jacket crowns will continue to do so, but the way is pointed out how any dentist, with a simple office equipment, may render the best service to a patient when a porcelain crown becomes necessary.

